# Memo1 binds reduced copper ions, interacts with copper chaperone Atox1, and protects against copper-mediated redox activity in vitro

**DOI:** 10.1073/pnas.2206905119

**Published:** 2022-09-06

**Authors:** Xiaolu Zhang, Gulshan R. Walke, Istvan Horvath, Ranjeet Kumar, Stéphanie Blockhuys, Stellan Holgersson, Paul H. Walton, Pernilla Wittung-Stafshede

**Affiliations:** ^a^Department of Biology and Biological Engineering, Chalmers University of Technology, 412 96 Gothenburg, Sweden;; ^b^Department of Surgery, University of Gothenburg, 413 45 Gothenburg, Sweden;; ^c^Department of Chemistry and Chemical Engineering, Chalmers University of Technology, 412 96 Gothenburg, Sweden;; ^d^Department of Chemistry, University of York, Heslington, York YO10 5DD, United Kingdom

**Keywords:** copper-binding protein, cancer, reactive oxygen species, Atox1, Memo1

## Abstract

The protein mediator of ERBB2-driven cell motility 1 (Memo1) is connected to many signaling pathways that play key roles in cancer. Memo1 was recently postulated to bind copper (Cu) ions and thereby promote the generation of reactive oxygen species (ROS) in cancer cells. Since the concentration of Cu as well as ROS are increased in cancer cells, both can be toxic if not well regulated. Here, we investigated the Cu-binding capacity of Memo1 using an array of biophysical methods at reducing as well as oxidizing conditions in vitro. We find that Memo1 coordinates two reduced Cu (Cu(I)) ions per protein, and, by doing so, the metal ions are shielded from ROS generation. In support of biological relevance, we show that the cytoplasmic Cu chaperone Atox1, which delivers Cu(I) in the secretory pathway, can interact with and exchange Cu(I) with Memo1 in vitro and that the two proteins exhibit spatial proximity in breast cancer cells. Thus, Memo1 appears to act as a Cu(I) chelator (perhaps shuttling the metal ion to Atox1 and the secretory path) that protects cells from Cu-mediated toxicity, such as uncontrolled formation of ROS. This Memo1 functionality may be a safety mechanism to cope with the increased demand of Cu ions in cancer cells.

## Significance

Since many proteins depend on copper ions for functionality, it is not surprising that cancer cells have a high demand for copper. Still, free copper ions are toxic as they can potentially catalyze the formation of harmful reactive oxygen species (ROS) upon coupling redox cycling between Cu(I) and Cu(II) with reduction of O_2_. Here, we investigated copper binding to Memo1, an oncogenic protein linked to cancer. We demonstrate that Memo1 coordinates reduced but not oxidized copper ions, thereby preventing the copper ions from acting as redox catalysts for ROS generation. As Memo1 is a putative target for the treatment of cancer, it is of importance to identify its binding partners (e.g., metal ions) and the functional consequences of such interactions.

Many proteins coordinate copper (Cu) ions to facilitate biological processes such as cellular respiration, protection against oxidative stress, biosynthesis of chemical messengers, modulation of connective tissue, and pigment construction (1–3). Many of these Cu-dependent activities exploit Cu redox cycling between oxidized (Cu(II)) and reduced (Cu(I)) forms. Since free Cu is toxic due to its redox activity that can damage biomolecules and generate reactive oxygen species (ROS), cells contain an elaborate network of Cu-transport proteins that facilitate specific and timely Cu delivery to target Cu-dependent proteins. Because of its role in many biological processes (4–6), Cu is also required in several distinguishing cancer phenomena (e.g., proliferative immortality, angiogenesis, metastasis), and cancer patients’ serum and tumors have increased Cu levels (7). For example, roles in cancer metastasis of the Cu-dependent proteins LOX (8), SPARC (9), and MEK1 (10) have been reported. Indeed, in this context, we recently showed that the Cu chaperone Atox1, which moves Cu(I) in the cytoplasm from the Cu importer Ctr1 to ATPases ATP7A and ATP7B in the Golgi network for loading of Cu-dependent enzymes in the secretory pathway, also promotes cancer cell migration (11). In accord, poor survival of breast cancer patients correlates with high Atox1 expression in the tumor (12).

The mediator of ERBB2-driven cell motility 1 (Memo1) is a protein connected to cancer progression that has been proposed as a cancer drug target (13–15). Its name comes from the fact that it was first identified in a proteomic screen of molecules that bound to the Erb-B2 receptor tyrosine kinase 2 (ERBB2): Memo1 was found necessary for efficient cell migration upon receptor activation (16). Accordingly, in our hands, silencing of the Memo1 gene in breast cancer cells reduced wound healing ability (a measure of cell migration; *SI Appendix*, Fig. S1). In addition to the context of breast cancer metastasis, it is clear that Memo1 has functions in additional cancer cell types, in developmental processes during embryogenesis, and in the homeostatic regulation of adult organ systems. In fact, Memo1 has been colloquially described as having a “Swiss Army knife”–like collection of functions and interaction partners (16).

**Fig. 1.**
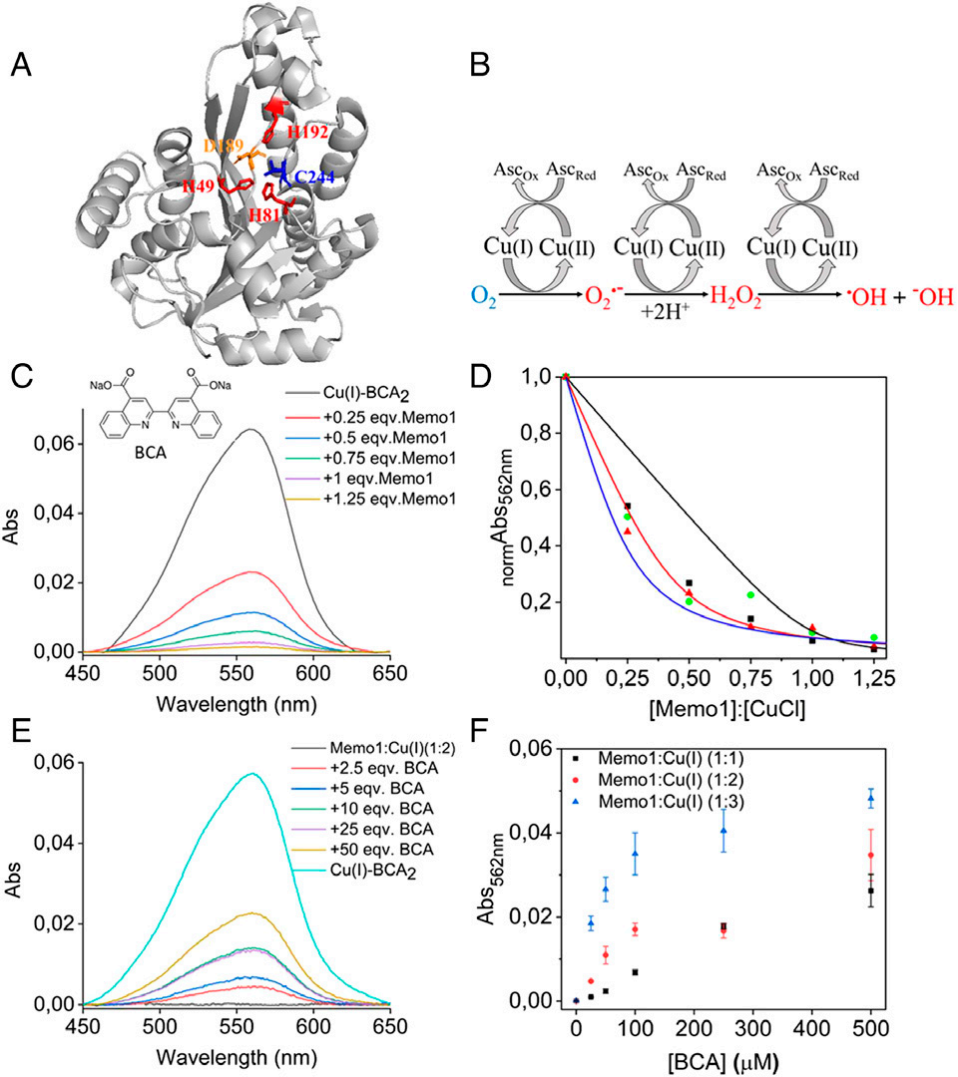
(*A*) Structure of Memo1 (PDB: 3BCZ), with the putative metal-binding site, side chains of His (49, 81, and 192), Asp (189), and Cys (244) in red, orange, and blue stick representation. (*B*) Redox cycling of Cu(II)/Cu(I) is fueled by reducing agents (here, ascorbate acid) in aerobic conditions that result in the production of toxic ROS (shown in red color) from nontoxic molecular oxygen, O_2_ (shown in blue), such as superoxide radicals (O_2_^•^**^−^**), hydrogen peroxide (H_2_O_2_), hydroxyl radicals (^•^OH), and hydroxide ions (^−^OH). (*C*) Cu(I)-BCA_2_ competition assay with Memo1 at strict anaerobic conditions. Absorption spectra of Cu(I)-BCA_2_ (10 µM) as a function of additions of Memo1 (0.25 to 1.25 molar ratio of Memo1 to Cu(I)). All of the spectra were analyzed after background subtraction. (*D*) Normalized absorbance at 562 nm plotted as a function of added Memo1 (x-axis given as Memo1 to Cu(I) ratio in the sample, Cu(I) concentration is 10 µM). Data from three independent experiments are included. Results for the best fits of Eq. **2** (*Materials and Methods*, *SI Appendix*) assuming one (black), two (red), or three (blue) independent Cu(I) site per Memo1 protein. The R^2^ values for fits assuming 1, 2, or 3 Cu(I) per protein are 0.83, 0.97, and 0.90, respectively. (*E*) Absorption spectra of 5 µM Memo1 premixed with 2:1 molar ratio of Cu(I) (10 µM) at anaerobic conditions, following the additions of BCA (2.5 to 50 molar ratio of BCA over Cu(I)). Cu(I)-BCA_2_ (10 µM) shown for reference. All of the spectra were analyzed after background subtraction. (*F*) Absorbance at 562 nm of BCA titrations (2.5 to 50 molar ratio of BCA over Cu(I)) to premixed samples of 10 µM Cu(I) mixed with 3.3 µM (3 Cu per protein), 5 µM (2 Cu per protein), and 10 µM (1 Cu per protein) Memo1. The error bars represent the SD for the average of three independent measurements.

The high-resolution structure of Memo1 reported in 2008 demonstrated a fold homologous to nonheme iron dioxygenases (17). In contrast to iron-binding nonheme dioxygenases, the putative metal-binding pocket of Memo1 includes three His (49, 81, and 192), one Asp (189), and one Cys (244) (instead of a Glu found in homologs) residue. Notwithstanding the capacity of these residues to coordinate Cu ion(s), no density was found in the crystal structure that would indicate a metal ion bound to Memo1 at this site (Fig. 1*A*). Subsequently, Memo1 was proposed to be a Cu(II)-dependent redox protein that promotes a more oxidized intracellular environment via production of ROS (18). The exchange of Glu for Cys in the putative metal-binding site in Memo1 supports a preference for Cu. For redox cycling, Cu(II) needs to be reduced to Cu(I), which in turn can activate molecular oxygen (O_2_) to produce the superoxide radical (O_2_^•−^) while returning to Cu(II) (Fig. 1*B*). (19, 20) Thus, for this activity, the protein needs to coordinate the Cu such that the reduction potential of any Cu(II) state is within the range of common in vivo reducing agents (e.g., glutathione). ROS can harm cellular components but also act as an important regulator of signaling pathways. ROS is elevated in tumor cells, promoting many cancer processes, but too-high levels of ROS can lead to cell death through various mechanisms (21). Because of the higher levels of Cu in cancer cells, arbitrarily released Cu ions may contribute to uncontrolled ROS generation, although the importance of Cu-generated ROS in cancer cells is not known. Since the previous work (16) was mostly performed in cell cultures and with excess Cu(II) added, several molecular questions about the putative Memo1-Cu interaction and link to ROS remain. Recently, an X-ray structure of Memo1 with a single Cu(I) coordinated to His49, His81, and Cys244 residues was deposited in the Protein Data Bank (PDB; 7L5C) (22). Although the metal occupancy in the structure was low, and the coordination geometry incommensurate with Cu-ligand distances (Cu…N > 2.6 Å) expected based on other Cu proteins and complexes, it supports the hypothesis that Cu (at least in the reduced state) can be coordinated by the protein.

Here, we have purified Memo1 and characterized its ability to bind Cu(II) and Cu(I) using a range of experimental methods in vitro at physiological conditions (pH 7.4). We find that the protein favors Cu(I) binding, and such an interaction protects toward Cu-mediated redox (ROS generating) activity in vitro. We also show that the Cu chaperone Atox1 can exchange Cu(I) with Memo1 in vitro, and that the two proteins exhibit a Cu-dependent interaction in surface plasmon resonance (SPR) experiments. Complementary proximity ligation experiments in MDA-MB-231 breast cancer cells imply interactions of Atox1 and Memo1 in living cells.

## Results

### Binding of Cu to Memo1 In Vitro.

Purified Memo1 is folded and monomeric (*SI Appendix*, Figs. S2 and S3*A*); it exhibits a thermal melting point above 50 °C at pH 7.4 (dependent on buffer and salt, *SI Appendix*, Fig. S3*B*). Because Memo1 was reported to bind Cu(II) (17), initially, we attempted to confirm this using spectroscopy (23, 24) and titration calorimetry (25) (*SI Appendix*, Fig. S4). However, no spectroscopic or energetic evidence for such an interaction was detected (using low micromolar concentrations of protein and Cu in stoichiometric or substoichiometric amounts). However, we noted a strong tendency for protein aggregation and precipitation as soon as one molar equivalent or excess Cu(II) ions were added. This behavior was also noted upon Zn(II) additions (*SI Appendix*, Fig. S5), and we attribute this to nonspecific metal interactions with the many His and Cys residues exposed on the protein surface (*SI Appendix*, Fig. S6) (26, 27). We speculate that Cu(II)-induced aggregation of Memo1 at nonreducing conditions may explain some of the results in the previous work (16).

We next turned to the assessment of possible Cu(I) binding to Memo1. Here, we used the bicinchoninic acid (BCA) ligand, a Cu(I) chelator, for competitive Cu(I) binding. BCA can form a chromophore, Cu(I)-BCA_2_ complex at 1:2 stoichiometry with high absorption at 562 nm (ε_562nm_(Cu(I)-BCA_2_) = 7,100 M^−1^ cm^−1^) (28). Titration of Memo1 to preformed Cu(I)-BCA_2_ at anaerobic conditions results in a decrease in absorption at 562 nm, which is indicative of Memo1 competing with BCA for Cu(I) (Fig. 1*C*). To determine the affinity and stoichiometry of the Memo1 interaction with Cu(I), we analyzed BCA titration data following equations outlined in the *Materials and Methods* section. For this, BCA was loaded with Cu(I) in a BCA/Cu(I) molar ratio of 2.5 at anaerobic conditions. This ensures that all Cu(I) is found in the 1:2 complex with BCA, with negligible amounts of the 1:1 complex. The Cu-loaded BCA was then titrated with Memo1 while keeping the concentrations of Cu(I) and BCA constant. The experimentally determined decrease in absorbance at 562 nm is plotted against the ratio of Cu(I) to protein in Fig. 1*D*. Immediately clear from inspection of the titration data is that Memo1 binds more than one Cu(I) per protein. Indeed, analysis of the data according to Eq. **2** (which includes the formation constant, β_2_ = 10^17.2^ M^−2^ for Cu(I)-BCA_2_ carefully determined by Xiao et al. (29)), assuming one Cu site per protein, gives a poor fit. In contrast, analysis assuming two independent Cu(I) sites per protein, with equal affinity, gives a much better fit to the data (Fig. 1*D*). To also assess the possibility of 3 Cu(I) binding per Memo1, we also fitted the data assuming such a stoichiometry, but this fit was worse than the 2 Cu(I) per protein assumption. Assuming two (independent, but equal in terms of affinity) Cu(I) sites per protein results in an affinity of each Cu(I) for Memo1 of 4 × 10^−15^ M. However, we cannot exclude that one binding site has a higher affinity and the other site, a lower affinity.

To address the stoichiometry of Cu(I) binding further, we performed the experiment the other way around (i.e., Memo1 was premixed with Cu(I) in 1:1, 1:2, and 1:3 Memo1 to Cu(I) ratios at anaerobic conditions, followed by BCA additions). An example of such a titration experiment for a 1:2 molar ratio of Memo1 to Cu(I) is shown in Fig. 1*E* (experimental data for 1:1 and 1:3 is shown in *SI Appendix*, Fig. S7). The appearing absorption at 562 nm was followed as a function of added BCA. These experiments show that BCA, when added in sufficient excess, can extract Cu(I) from Memo1. The BCA titration profiles look similar for BCA added to 1:1 and 1:2 Memo1 to Cu(I) premixed samples (Fig. 1*F*). Clearly, not much Cu(I)-BCA_2_ is formed in the first few BCA additions. However, for the 1:3 Memo1 to Cu(I) premixed sample, the first addition of BCA (2.5 equiv of BCA to Cu(I)) showed an increase in absorption at 562 nm that corresponds to one-third of the total Cu(I). This suggests that one-third of the total Cu(I) in this sample is free in solution and directly available for BCA binding. The remaining Cu (two-thirds of total) is thus bound to Memo1 (which is present at one-third concentration relative total Cu) and needs excess BCA to be removed from the protein.

To directly confirm binding of Cu ions to the protein, Memo1 samples purified from Cu(I)-BCA_2_ titrations (at conditions in which spectroscopy indicated Cu(I) loaded to Memo1) were submitted for inductively coupled plasma-mass spectrometry (ICP-MS) analysis (*SI Appendix*, Table S1). The ICP-MS results for dialyzed protein demonstrate that upon mixing with Cu(I)-BCA_2_, Cu becomes bound to Memo1. We also tested whether the Memo1 protein itself could possibly act as an antioxidant and reduce Cu(II) to Cu(I). However, the addition of excess BCA (sufficient based on above experiments to pull out Cu(I) from Memo1) to a mixture of Memo1 and Cu(II) did not result in any visible absorption from the formation a Cu(I)-BCA_2_ complex. Taken together, the BCA experiments clearly indicate that two Cu(I) coordinate per Memo1 molecule.

### Memo1 Protects Cu from Redox Cycling In Vitro.

**Fig. 2.**
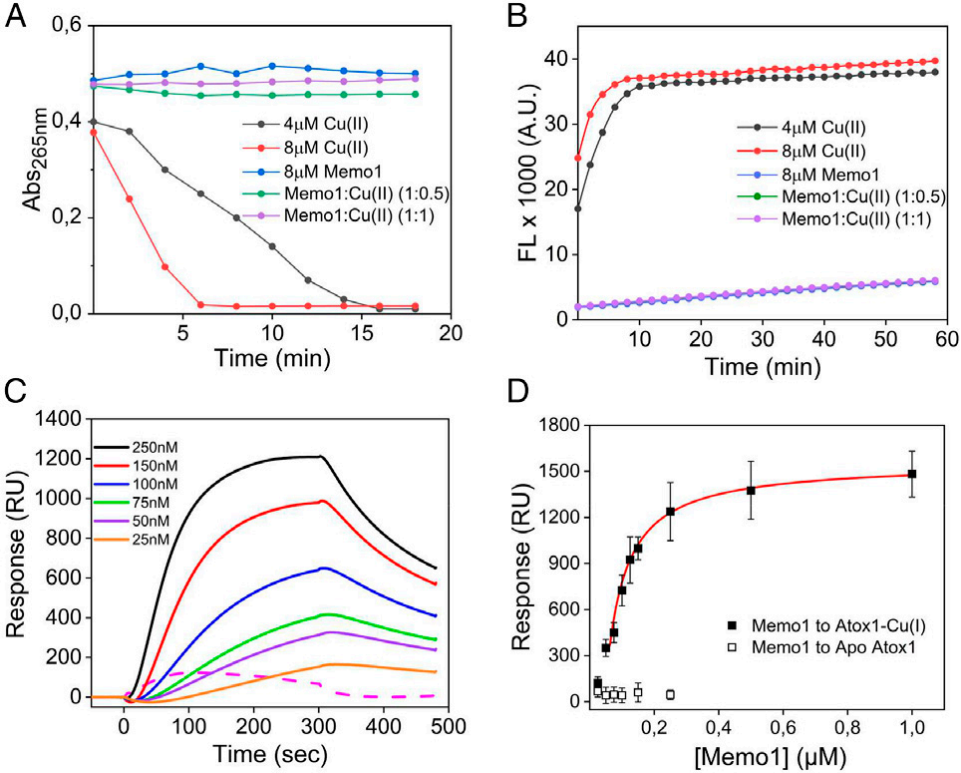
Role of Memo1 in redox reactions (*A* and *B*) and binding to a Cu chaperone (*C* and *D*). (*A*) Ascorbic acid oxidation as a function of time in the presence of Cu(II) and dioxygen without and with the addition of Memo1 at molar ratios of 1:0.5 and 1:1 Memo1 to Cu(II). (*B*) Cu-catalyzed H_2_O_2_ production in the presence and absence of Memo1 monitored via fluorescence of resorufin (excitation λ = 571 nm; emission λ = 585 nm) as a function of time, in the presence of ascorbate, Cu(II), and dioxygen. (*C*) Detection of Memo1 binding to Cu(I)-loaded Atox1 by SPR (solid curves). The dashed curve shows 250 nM Memo1 injected onto apo-Atox1 surface. (*D*) The affinity was obtained by fitting of the binding levels at the end of the injection versus Memo1 concentration curves to a 1:1 binding model using evaluation software provided by the manufacturer (GE Healthcare). Data are representative of three independent experiments.

Reducing agents such as ascorbic acid can initiate redox cycling of Cu by reducing Cu(II) to Cu(I). In the presence of oxygen, Cu(I) can be reoxidized to Cu(II), which releases an electron that reacts with molecular oxygen to form a superoxide radical. Further Cu redox cycles, using the reducing agent to regenerate Cu(I), result in the production of hydrogen peroxide (H_2_O_2_), hydroxyl radicals (^•^OH), and hydroxide ions (^−^OH) (Fig. 1*B*) (30). Using ascorbic acid as the reducing agent, Cu redox cycling can be followed by absorption changes at 265 nm (21), which reports on oxidation of ascorbic acid (31). Cu(II) alone consumes ascorbate rapidly due to efficient redox cycling of Cu in solution, and this is observed as a fast decrease in absorption at 265 nm with time (Fig. 2*A*). In the presence of Memo1 (1:0.5 and 1:1 molar ratio of Memo1 to Cu(II)), redox cycling of Cu (i.e., oxidation of ascorbic acid) is inhibited. Thus, it appears that ascorbic acid reduces Cu(II) in solution to Cu(I), which then binds to Memo1 and in this complex the Cu(I) is inhibited from reoxidation to Cu(II).

To confirm this result, we carried out the Amplex Red assay to detect Cu-induced H_2_O_2_ generation. The Amplex Red assay detect H_2_O_2_ via oxidation of 10-acetyl-3,7-dihydroxypenoxazine (Amplex Red), a reaction that is catalyzed by horseradish peroxidase (HRP). In the presence of H_2_O_2_, the nonfluorescent Amplex Red is oxidized to the red fluorescent product resorufin (32). In accord with the ascorbate oxidation results, mixing of ascorbate and Cu(II) in the Amplex Red assay rapidly generated resorufin (Fig. 2*B*). However, upon addition of Memo1 in the reaction mixture (0.5 and 1 Cu(II) per Memo1), there was no generation of H_2_O_2_ detected. (We could not test higher Cu to Memo1 ratios in these assays as the protein started to precipitate within the experimental time frame.) Taken together, these redox cycling measurements support that Memo1 binds Cu(I), and, in so doing, the metal ion is protected from redox reactions and thus ROS production.

### Memo1 Interaction with Cu Chaperone Atox1 In Vitro.

There is no free Cu in cells at normal conditions (33), so if Memo1 is a Cu-binding protein in vivo, the metal ion may be delivered to Memo1 by another protein. A putative candidate is Atox1, the cytoplasmic Cu chaperone that delivers Cu(I) to Cu-dependent enzymes in the secretory pathway (34). We therefore tested whether Cu(I)-loaded Atox1 would interact with Memo1 using SPR (Fig. 2*C*) (35). Such analysis demonstrated an interaction with low-micromolar affinity of 2.1 × 10^−7^ M (Fig. 2*D*). Atox1 without Cu(I) (apo-Atox1) did not interact with Memo1, suggesting that the interaction is Cu(I) dependent.

**Fig. 3.**
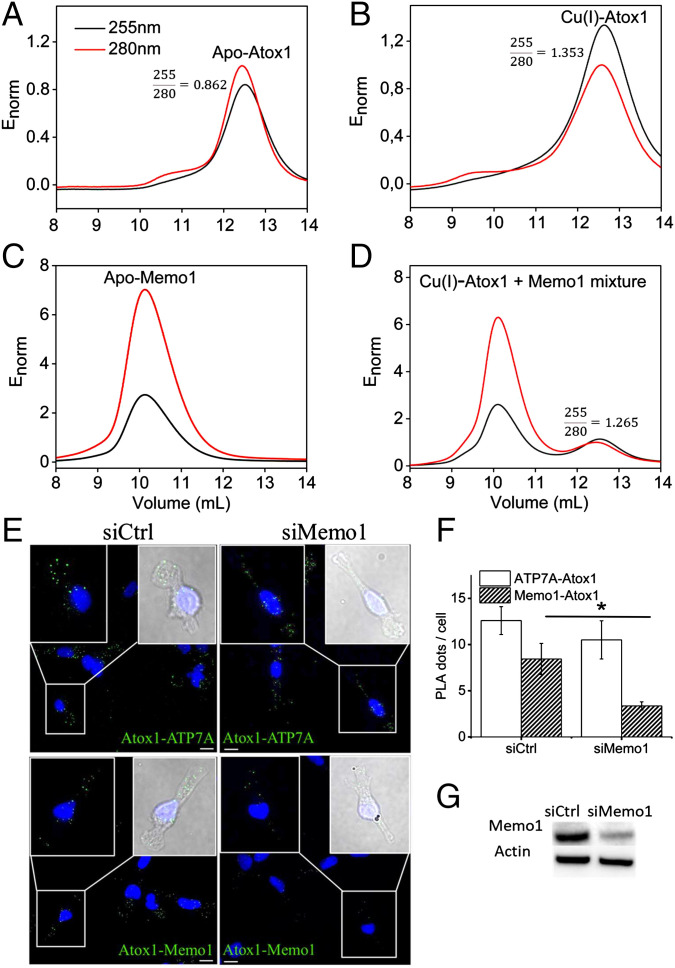
Cu transfer between Memo1 and Atox1 in vitro and their proximity in cancer cells. Cu transfer from Atox1 to Memo1 probed by SEC (*A*–*D*). Elution profiles from SEC representing absorbance at 255 nm (black) and 280 nm (red). All of the elution profiles are normalized (E_norm_) by Atox1, so the maximum absorbance of Atox1 at 280 nm is 1. (*A*) 50 µM apo Atox1. (*B*) Atox1 was loaded with Cu(I) in a 1:1 ratio. (*C*) 20 µM apo Memo1. (*D*) Mixture of Cu(I)-Atox1 and apo-Memo1. In mixing experiments, Atox1 (50 µM) was first loaded with Cu(I) in a 1:1 ratio and, afterward, holo-Atox1 was mixed with apo Memo1 (20 µM) followed by SEC analysis. 255/280 nm absorption ratios are reported for the Atox1 peak in three panels. *SI Appendix*, *Fig. S8* also includes more Memo1 traces. PLA results for Memo1-Atox1 and ATP7A-Atox1 proximities in MDA-MB-231 breast cancer cells (*E*–*G*). (*E*) Fluorescence microscopy images and the overlay of bright-field images (blue, DAPI, indicating the nucleus, and green, PLA dots) to visualize the cells and perform quantification, illustrating PLA results for ATP7A and Atox1 (positive control; top images) and for Memo1 and Atox1 (bottom images) with PLA dots in green (GFP) and cell nuclei in blue (DAPI). Scale bars indicate 10 µm. (*F*) PLA dots were counted in cells (an average of 68 cells analyzed per condition). Error bars indicate SD of the means (*n* = 68). (*G*) Western blot results of Memo1 knockdown (siMemo1) and control cells (siCtrl).

We also analyzed Cu(I) transfer from Atox1 to Memo1 using size exclusion chromatography (SEC) separation combined with dual wavelength absorption detection, as previously performed for Atox1 and metal-binding domains in ATP7B (36, 37). Using the absorption ratio 255:280 nm as the probe for the Cu-loading level of Atox1 (37), we found that after mixing with Memo1 (apo-Memo1 mixed with Cu-Atox1), the SEC-separated Atox1 peak contained less Cu than just SEC analysis of Cu-loaded Atox1 (Fig. 3*A*–*D*). Based on the 255:280 nm ratio change (values noted in Fig. 3 panels), roughly 15 to 20% of the Atox1 has become apo-protein after being in the mix with Memo1. This suggests that the “missing” Cu has been transferred to Memo1. The 255:280 nm absorption ratio cannot be used to determine Cu loading of Memo1 due to its high content of aromatic residues, but the presence of Cu can be detected via BCA titration (formation of Cu(I)-BCA_2_ giving visible absorption) to the Memo1 elution peak (*SI Appendix*, Fig. S8). To directly prove that the Cu that left Atox1 had transferred to Memo1, we analyzed the Memo1 elution peak after SEC separation of the mixture using excess BCA additions (*SI Appendix*, Fig. S9). Such BCA titrations confirmed the presence of Cu in the Memo1 elution peak (via visible absorption from Cu(I)-BCA_2_), although we could not quantify the total amount of Cu as Memo1 precipitates when too much BCA is added. In a control experiment, we detected no Cu in the Memo1 peak when apo-Atox1 was mixed with apo-Memo1 (*SI Appendix*, Fig. S9).

The estimated dissociation constant of Memo1 for Cu(I) is approximately 10^−15^ M when assuming two identical and independent sites (Fig. 1*D*). The Cu(I) affinity for Atox1 is reported to be approximately 10^−17^ M (29), and, using the same conditions as for Memo1, we confirmed the Cu(I) affinity for Atox1 to be approximately 10^−17^ M at our conditions (*SI Appendix*, Fig. S10). Thus, based on simple thermodynamics, the Cu(I) should stay in Atox1 upon mixing with Memo1. However, as we know from the SPR data that the proteins can form a heterocomplex, Atox1-Cu(I)-Memo1, it is the Cu(I) coordination in the complex that defines which protein receives the metal ion upon flow perturbation. It is also possible that Memo1 binds the two Cu(I) ions with different affinities of which one is comparable to the Atox1 Cu(I) affinity. The binding analysis in Fig. 1*D* cannot exclude that one Cu(I) binds tighter. The SEC analysis of the Cu-Atox1 and Memo1 mixture did not reveal a heterocomplex of the proteins that survived elution from the column. Thus, in contrast to Cu transfer between Atox1 and metal-binding domain 4 of ATP7B (37) but similar to the reaction between Atox1 and metal-binding domains 5 and 6 of ATP7B (36), the Cu-dependent interaction between Atox1 and Memo1 observed by SPR does not survive SEC. Taken together, Atox1 can transfer Cu(I) to Memo1, in addition to forming a Cu-dependent protein-protein complex in vitro. However, the affinity data imply that the preferred Cu(I) transfer direction (depending on solution conditions and concentrations) may be from Memo1 to Atox1.

### Proximity of Memo1 and Atox1 in Breast Cancer Cells.

To link the in vitro interaction data to cells, we used a proximity ligation assay (PLA) (11) to test for Atox1 and Memo1 proximity in breast cancer cells. The PLA assay does not detect direct protein interactions but indicates whether proteins are in less than 40 nm proximity of one another (11). In this way, we found the proximity of Atox1 and Memo1 in MDA-MB 231 cells (Fig. 3*E*). As a positive biological control, the proximity of Atox1 and ATP7A in the cells was detected, which has also been reported before (38). Silencing of Memo1 protein expression in the cells (to approximately 57%) resulted in an approximately 60% decrease in the number of PLA dots per cell, implying that the analysis is specific (Fig. 3*F* and *G*) (*Materials and Methods* and *SI Appendix*, Fig. S11 for control experiments).

## Discussion

Memo1 is an essential protein reported to have many interaction partners in different cellular signaling pathways (16). It is a key player in cancer progression, with activities that promote cell migration and metastasis (13, 14). Initially, Memo1 was shown to be an interaction partner with the tyrosine kinase ERBB2 receptor, but later work showed the protein to also play roles in signaling pathways of Fc fragment of immunoglobulin G receptor, insulin-like growth factor 1 receptor, estrogen receptor, platelet-derived growth factor receptor, and sphingosine 1-phosphate/S1P receptor families (16, 39). Downstream interaction partners include RhoA, mDia1, and cofilin, which provide links to the remodeling of the actin network and, thus, cell migration (40, 41). The structure of Memo1 revealed a fold homologous to bacterial dioxygenases (17), but no metal density was detected, and the putative metal site included a Cys residue not found in the homologs. Subsequent in vitro studies suggested instead that the “metal site” interacted with a peptide harboring the phosphorylated tyrosine segment of the C terminus of ERBB2; however, strong peptide binding required low pH and thus His protonation (42). More recently, Memo1 was proposed to bind Cu(II), and, via redox cycling, to stimulate production of ROS (18). Since the latter study was performed with excess Cu(II) and mostly using cell culture experiments, free Cu ions not bound to Memo1 could have played an inadvertent role in the generation of ROS. As such, we decided to carefully investigate putative Cu binding to Memo1 using purified protein and stoichiometric titrations. In contrast to earlier proposals, we found no evidence of Cu(II) binding to Memo1 at micromolar concentrations. Instead, the protein binds two Cu(I) ions per protein, and this interaction protects the metal ions from participating in redox activity. The Cu chaperone Atox1, when Cu(I) loaded, interacts with Memo1 with micromolar affinity, and the formation of this complex can result in Cu(I) transfer from Atox1 to Memo1. Although only indirect evidence of in-cell interaction, proximity studies in breast cancer cells show Memo1 and Atox1 to be spatially close.

Our results raise several questions about the role of Memo1 in cancer cells: how are two Cu(I) ions coordinated in Memo1? Is it a di-nuclear Cu(I) center in the expected metal binding site? A di-nuclear Cu(I) site is found in hemocyanins, tyrosinases, and catechol oxidases and involves mostly His coordination (43, 44). However, in those proteins, the di-nuclear Cu site is redox active and can bind oxygen. We do not find any evidence of redox activity or Cu(II) coordination in Memo1. In the Memo1 structure deposited in 2022 (7L5C, obtained at pH 5), one Cu(I) is modeled in the metal binding site as coordinating to side chains of His49, His81, and Cys244 (22). However, the Cu-ligand bond lengths in this structure are too long when compared to expected Cu-ligand distances, and it may instead, at physiological pH, be a binuclear Cu site that involves additional His side chains. Notably, near the modeled Cu site is a water ion coordinated by three His side chains (His12, His82, and His131), which, if the pH is raised (the crystal structure was determined at pH 5), may coordinate another Cu(I) (*SI Appendix*, Fig. S12). Alternatively, there may be two well-separated Cu(I) binding sites, with one metal ion in the expected metal binding site and the other Cu ion in another part of the protein, as there are many additional His and Cys residues throughout the protein.

Another question that arises is whether Cu(I) binding to Memo1 has functional relevance? One possibility is that by chelating Cu(I), Memo1 protects cells from unwanted Cu-mediated redox reactions that can harm biomolecules and generate uncontrolled ROS. It is well known today that the production of ROS is elevated in cancer cells because of increased metabolic activity, gene mutations, and hypoxia. Moderate increases in ROS promote cancer progression (21), but there must be tight control and additional antioxidant activities have been found to be essential for tumorigenesis (21). Since cancer cells have increased Cu levels (7), and free Cu ions may promote ROS formation, unless controlled, Memo1 may act as a Cu(I) chaperone in cancer cells. Since the affinity of Cu(I) appears higher for Atox1 than for Memo1, Memo1 may channel Cu(I) to Atox1 as a way to remove dangerous Cu(I) ions into the secretory pathway, where excess Cu can be exported. In contrast to the previous report on Memo1 (18) and other cancer cell lines studies, but in alignment with our results, work in *Caenorhabditis elegans* and mice showed that the loss of Memo1 was associated with an increase in ROS within the organism (16). An additional possibility is that interactions between Memo1 and partners in various signaling pathways are tuned, or turned on/off, by the presence or absence of Cu(I) in Memo1. For example, at low pH, where His residues are protonated, Cu(I) will not be bound, but the interaction with the ERBB2 peptide is favored. Further studies from both molecular-mechanistic and functional-dysfunctional angles are needed (work in progress).

## Materials and Methods

Memo1 and Atox1 proteins were purified by heterologous expression in BL21(DE3) cells. BCA competition was used at anaerobic conditions to determine Cu(I) binding to Memo1. Ascorbate oxidation and the Amplex Red Assay were used for redox activity tests. Memo1-Atox1 binding was assessed by the SPR method, involving immobilized His-tagged Atox1. Memo1 was silenced in MDA-MB-231 breast cancer cells by transient transfection with siRNA. Further details on these experiments are provided in the *SI Appendix*. Also, circular dichroism, isothermal titration calorimetry, SEC, ICP-MS, western blotting, and PLA experiments as well as equations for fitting of Cu(I)-binding data are described in the *SI Appendix*.

## Supplementary Material

Supplementary File

## Data Availability

All of the study data are included in the article and/or *SI Appendix*.
