# Supplementary material for: Memo1 binds reduced copper ions, interacts with copper chaperone Atox1, and protects against copper-mediated redox activity in vitro
Source: Proc Natl Acad Sci U S A. 2022 Sep 6;119(37):e2206905119. doi: 10.1073/pnas.2206905119 (PMC9477392; doi:10.1073/pnas.2206905119)
Supplement: Supplementary File [file pnas.2206905119.sapp.pdf]

PNAS

Supplementary Information for

Memo1 binds reduced copper ions, interacts with copper chaperone Atox1, and protects against copper-mediated redox activity *in vitro*

Xiaolu Zhang[1], Gulshan R. Walke[1], Istvan Horvath[1], Ranjeet Kumar[1], Stéphanie Blockhuys[2], Stellan Holgersson[3], Paul H. Walton[4], Pernilla Wittung-Stafshede[1]*

Corresponding author: Pernilla Wittung-Stafshede
Email: pernilla.wittung@chalmers.se

This PDF file includes:

Materials and Methods
Figures S1 to S12
Table S1
SI References

Materials and Methods

Expression and purification of Memo1. Memo1 was purified by heterologous expression of pET28a construct having the Memo1 gene into BL21 (DE3) (Novagen) cells. The bacteria were first grown to an OD_{600} of 0.6 in Luria broth (LB) containing 50 µg/ml Kanamycin at 37°C and then induced with 1mM IPTG (isopropyl β-D-1-thiogalactopyranoside) and grown overnight at 25°C post induction. The cells were harvested and lysed by sonication on an ice bath in 20mM Tris-HCl buffer pH 8.0 and 10 percent glycerol in the presence of a protease inhibitor cocktail (Roche). The lysate was then treated with Universal Nuclease (Pierce) at RT for 30 min followed by centrifugation at 16000 g for 30 min. The supernatant was then filtered through a 0.2µm filter and then loaded onto a pre-equilibrated 5ml HiTrap Q FF anion exchange column (GE Healthcare). The flowthrough was then loaded onto a 5ml His trap column (GE Healthcare). The protein was eluted by using 20mM Tris-HCl buffer pH 8.0 containing 250mM Imidazole after an initial wash with 10mM Imidazole. 1mM TCEP (tris (2-carboxyethyl) phosphine) and 5 percent Glycerol were added soon after the elution. The protein was then loaded onto a pre-equilibrated Hiload 16/600 Superdex 75 pg column (GE Healthcare) with 20mM HEPES containing 200mm NaCl. The eluted protein was run on a 4-12% SDS-PAGE and fractions containing the protein were pooled and concentrated with Amicon Ultra-15 10K centrifugal filter units (Millipore). The protein identity was further confirmed by Western blot using an antibody against Memo1 protein. The concentration of purified Memo1 was determined using ε_{280nm} = 52830 M^{-1} cm^{-1} and snap-frozen in liquid nitrogen and stored at -80°C.

Expression and purification of Atox1. Heterologous expression and purification of Atox1 with an N-terminal his tag (including a caspase cleavage site) in the pET-3a plasmid was performed by transforming the plasmid BL21(DE3) (Novagen) cells. The bacteria were first grown to an OD_{600} of 0.6 in Luria broth (LB) containing 100 µg/ml carbenicillin at 37°C and then induced with 1 mM IPTG (isopropyl β-D-1-thiogalactopyranoside) and grown overnight at 25°C post-induction. The cells were harvested and lysed by sonication on an ice bath in Buffer A (20mM Tris-HCl pH 8.0, 20mM imidazole) in the presence of a protease inhibitor cocktail (Roche). The lysate was centrifuged at 15000 g for 30 min. The supernatant was then filtered through 0.2µm filter and loaded onto a pre-equilibrated 5 ml HisTrap FF (GE Healthcare) IMAC column. The Atox1 protein was eluted by a linear gradient with 500mM Imidazole in 20mM Tris-HCl buffer pH 8.0. The eluted protein was run on a 4-12% SDS-PAGE and fractions containing the protein of interest were pooled and concentrated with Amicon Ultra-15 3K centrifugal filter units (Millipore). The concentrated protein was loaded and retrieved from a pre-equilibrated Hiload 16/600 Superdex 75 pg column (GE Healthcare) with 20mM Tris-sulfate buffer pH 7.4. The wild-type Atox1 (without His tag) was expressed and purified as in the previously published method (1) with small alterations. The concentration of Atox1 protein was determined using ε_{280nm} = 2980 M^{-1} cm^{-1}. For the binding experiments, the apo form of Atox1 was assured by treating it with an excess of EDTA and DTT and the final protein was retrieved by loading it onto a pre-equilibrated Superdex S75 10/300 GL

with 25mM HEPES and 200mM NaCl. Cu-loading of Atox1 was performed by 0.9 equimolar $CuCl_2$ in the presence of 5 times excess DTT.

Circular dichroism (CD). Thermally-induced unfolding experiments of 5µM Memo1 were performed in 20mM MES at pH 5.5, 50mM NaP at pH 7.4, 25mM HEPES, pH 7.4, and 50mM Tris pH 8.0, all with NaCl as indicated in figure legends. Full far-UV CD spectra of Memo1 were collected before thermal experiments at a single wavelength. In thermal experiments, CD was monitored at 222nm (1°C/min; 1mm cell). The data were fitted to two-state transitions with T_m defined as the midpoint temperature. As Memo1 thermal unfolding was irreversible, the T_m values are only apparent. The visible CD was probed in a 1 cm cell. CD was monitored by an Applied Photophysics Chirascan instrument.

Size-Exclusion Chromatography (SEC). SEC was performed with a Superdex 75 10/300 analytical column (volume of 24 mL) on an NGCTM purifier (Bio-Rad) at 10°C. The column was pre-equilibrated with 25mM HEPES pH 7.4 with 200mM NaCl. The protein samples were prepared in the same buffer. The elution profiles of protein samples were monitored using dual-channel absorption detection at 255 and 280 nm. A range of experiments was performed at each condition: mixtures of apo-proteins, individual apo-proteins, Cu(I)-loaded Atox1, reaction mixtures (apo-Memo1 and Cu(I)-loaded Atox1; 30 min incubation at 4°C before loading). Initial Atox1 and Memo1 concentrations were 50 and 25µM.

Surface plasmon resonance (SPR) assay. For binding studies, His-tagged Atox1 was immobilized on a Biacore NTA chip (Cytiva, Uppsala, Sweden) using Ni chelation. Cu-loading of Atox1 was performed prior to attachment to the chip by adding equimolar $CuCl_2$ in the presence of 5 times excess DTT. Background correction was done by subtracting the signal from the flow channel where only Ni^{2+} was immobilized. Measurements were performed on a Biacore X100 system (Cytiva, Uppsala, Sweden) in multicycle mode at 10µl/min flow rate: each cycle consisted of an immobilization step (5µM Atox1, 3 min) after 10 min of baseline stabilization followed by the injection of Memo1 (5 min) in the desired concentration; after 2 min of dissociation time, the surface was regenerated by the injection of 0.35mM EDTA. For each measurement, the immobilization level for Cu- or apo-Atox1 was around 400 RU after the 10 min stabilization period. The immobilization level varied by less than 5% between the cycles within each measurement series. The running buffer was HBS-P (10mM HEPES, 150mM NaCl supplemented with 0.002% P20 detergent) (Cytiva, Uppsala, Sweden). Prior to use, all proteins were gel filtered into HBS buffer using a Biorad Enrich 70 column (BioRad). The affinity was obtained by fitting of the binding levels at the end of the injection vs Memo1 concentration curves to a 1:1 binding model using evaluation software provided by the manufacturer (GE Healthcare).

Isothermal titration calorimetry (ITC). ITC experiments were performed with an ITC200 (MicroCal) In a typical run, 25 automated injections of 1.65µl $CuCl_2$ with 180s breaks in between injections were made at 25°C and 600 rpm stirring speed in low feedback mode. The cell protein

concentration was 20μM, while the $CuCl_2$ concentration in the syringe was 500μM. The buffer was 25mM HEPES, 200mM NaCl at pH 7.4. The buffers for the syringe Cu sample and the cell protein sample were identical. Data integration, fitting and evaluation were performed using the software Origin^{TM} 7 with the ITC200 plugin provided by MicroCal/GE Healthcare.

Cu(II) titration to Memo1 solution. 10μM Memo1 in 25mM HEPES pH 7.4, 200mM NaCl was titrated with 1mM $CuCl_2$ solution in a 1 cm cell. Spectra from 200 to 800 nm were measured on a UV-visible spectrophotometer (Cary 3500).

BCA competition assay. Bicinchoninic acid disodium salt (BCA) was purchased from Sigma-Aldrich. 100mM BCA solution was prepared in MQ water. The CuCl stock solution (0.5 mM) was prepared in MQ water containing 1mM of ascorbic acid to make sure there is no possible Cu(I) oxidation. HEPES buffer and MQ water were kept overnight in glove box (Coy2, N_2 gas) prior stock preparations. All the stocks used were freshly prepared and the experimental additions were carried out in the glove box. For each data point in Memo1 titrations to Cu(I)-BCA_2, a sample was prepared with 50μM BCA, 20μM CuCl, and Memo1 of different concentrations, 10μM CuCl and Atox1 of different concentrations. Memo1 or Atox1 was always added last, then samples were transferred from the glove box to the spectrophotometer. Absorbance was measured from 200 to 800 nm, and the Cu(I)-BCA_2 absorbance at 562 nm was plotted to determine the affinity of Memo1 or Atox1 (P) for Cu(I) (metal ion, M) according to (Eq. 1). BCA is used as the competing ligand (L) for Cu(I).

$$P + ML_2 \rightarrow MP + 2L \quad (1)$$

As the complex of two BCA molecules with one Cu(I) ion gives a high-intensity absorption band at 562 nm (ε_{562nm}(Cu(I)-BCA_2) =7100 $M^{-1}cm^{-1}$), changes in the Cu(I)-BCA_2 complex concentration due to competition with another ligand (here Memo1 or Atox1) for Cu(I) can be detected by absorption changes. Xiao et al. applied this assay to various Cu(I)-binding proteins effectively (2). To ensure that all Cu(I) is found in the chromophoric 2:1 complex with BCA, with negligible amounts of the 1:1 complex, we used a 2.5 molar ratio of BCA to Cu(I). The Cu(I)-loaded BCA is then titrated with Memo1 or Atox1 (P in Eq.1) while keeping the concentrations of Cu(I) and BCA constant. As increasing amounts of Memo1 or Atox1 will compete for Cu(I), the concentration of the Cu(I)-BCA_2 complex decreases. The experimentally determined decrease in absorbance at 562 nm is plotted against the ratio of Memo1 to Cu(I). Eq. 2 (3) can be used to fit the experimental data:

$$K_D\beta_2 = \frac{([P]/[MP])-1}{\{([L]_{total}/[ML_2])-2\}^2[ML_2]} \quad (2)$$

where [L] total is the BCA concentration and [P] is the total Memo1 or Atox1 concentration. [MP] and [ML_2] are calculated as normalized absorbance changes upon adding Memo1(abs_{562}) or Atox1(abs_{562}) relative to the value without Memo1($abs_{562,0}$) or Atox1(abs_{562}):

$$[MP] = [M](1 - abs_{562}/abs_{562,0}) \quad (3)$$

$$[ML_2] = [M](abs_{562}/abs_{562,0}) \quad (4)$$

To obtain the dissociation constant, K_D, for Cu(I)-Memo1 or Cu(I)-Atox1 the determined value, $\beta_2 = 10^{17.2}$ M^{-2}, was used as the formation constant for Cu(I)-BCA_2 (2).

As the experimental data indicated more than one Cu(I) per Memo1, we analyzed the data using the above equation for the case of 2 Cu(I) sites and 3 Cu(I) siter per Memo1. For independent and equal sites, we can then use the same equation and simply increase the fictive protein concentration to represent the number of sites present in each scenario.

For the BCA experiments starting with Cu(I) mixed with Memo1, samples were prepared with 10µM CuCl, and 3.3, 5 and 10µM Memo1 (in different experimental sets) and BCA of different concentrations were added (from 2.5 to 100-fold). BCA was always added last, samples were transferred from the glove box to the spectrophotometer. Absorbance was measured from 200 to 800 nm, and the absorbance at 562 nm was plotted as a function of added BCA. All BCA titrations were performed numerous times and the data shown in Figures comes from three independent experiments at all conditions.

BCA additions to SEC eluted Memo1 (Figs. S8 and 9). Eluted Memo1 (in various mixtures, see figure legends) was collected after SEC and the protein concentration determined. 1mM ascorbate acid were added to prevent Cu(I) oxidation and then the sample was titrated with excess BCA (from 1mM to 12mM). Absorbance was measured from 450 to 650 nm for the appearance of Cu-BCA_2 signal. All BCA titrations were performed numerous times and the data shown comes from three independent experiments at all conditions.

Inductively coupled plasma mass spectrometry (ICP-MS). 10µM Memo1 solution (500µL) was mixed with Cu(I)-BCA_2 complex in 1:0.5 and 1:1 Memo1 to Cu ratios. After dialysis for 3 h at 4°C against 25mM HEPES buffer (0.5L), 0.2M NaCl, pH 7.4, samples were analyzed for Cu with ICP-MS. Before the analysis, the samples were diluted with 0.5M HNO_3 (Merck, Suprapur). Four standard solutions (0, 1, 10 and 100 ppb Cu) were prepared from 10ppm certified elemental standards in 2% HNO_3 (CPA Chem) by utilizing corresponding volumes of sample blank solution and 0.5M HNO_3. To determine Cu concentrations in a blank sample, a corresponding blank-free standard series was also made. The ICP-MS instrument (Thermo iCAP Q) was run in standard mode for five consecutive readings at the mass of 65 for Cu (to avoid the common $ArNa^+$ interference at mass 63) from which the average concentrations with standard deviation were calculated. The instrument sensitivity drift during the analysis was monitored and compensated for with the addition of internal standards of 2ppb Sc and In. Sample concentrations were calculated from the blank-corrected standard curves, and blank concentration was calculated from the difference in intercept between blank-corrected and blank-free standard curves. Typical instrumental detection limit was 0.01ppt (at 3σ confidence level).

Ascorbate oxidation assay. Fresh 1mM L-ascorbate acid (Sigma) solution was prepared just before each assay. 8µM Memo1 in 25mM HEPES pH 7.4, 200mM NaCl was mixed with 35µM

ascorbate acid and 0, 4 or 8µM Cu(II) was added, followed by absorption spectra detection (200-800 nm) every 2 mins for 20 min (1 cm cell, room temperature, Cary 3500 spectrophotometer).

AmplexTM red assay. The Amplex™ Red Hydrogen Peroxide/Peroxidase Assay Kit (ThermoFisher) was used according to the manufacturer's instructions in 96 well Corning® Nonbinding Surface (NBS™) Microplates. 100µL samples were prepared with 50µM ascorbate acid, 50µM AmplexTM red, 3.55 U/Ml horseradish peroxidase with 8µM Memo1 and with 0, 4 or 8µM Cu(II). Fluorescence was monitored using FLUOstar OPTIMA microplate reader (excitation-544nm, emission- 590nm).

Cell lines and siRNAs. The human breast cancer cell line MDA-MB-231 was obtained from American Type Culture Collection (LGC standards). The cells were maintained at 37℃ and 5% CO_2 in Dulbecco's Modified Eagle Medium (Gibco), supplemented with 10% heat-inactivated fetal bovine serum (FBS) (Invitrogen). Passage number was kept below 20, and the cells were tested for mycoplasma periodically. Control (siCtrl) and Memo1-silenced (siMemo1) MDA-231 cells were generated by transient transfection with AllStars negative control siRNA or a pool of 4 different siRNA Memo1 (Gene Solution siRNA, Qiagen), respectively, using Lipofectamine RNAiMAX transfection reagent (Invitrogen).

Western blotting. Proteins were extracted by lysis buffer containing 50mM Tris HCl, 150mM NaCl, 1mM EDTA, 1% Triton and 100U/mL protease inhibitor cocktail (Roche). Protein concentrations were measured by the colorimetric BCA protein assay (Alfa Aesar). Equal amounts of protein for each sample were subjected to SDS/PAGE and transferred onto PVDF membranes (Bio-Rad). Membranes were first probed with primary antibodies rabbit anti-Memo1 antibody (sigma); rabbit anti-beta actin antibody (ab8227, Abcam) and subsequently probed with HRP-conjugated goat anti-rabbit secondary antibody (Sigma). Protein bands were detected using the ECL plus Western blotting detection system (GE Healthcare) and ChemiDoc™ touch imaging system (Bio-Rad).

In situ proximity ligation assay (PLA). We followed the previously published method for PLA assay (4). PLA was performed on fixed MDA-231 cells using the Duolink® Detection Reagents Green kit (Sigma-Aldrich) according to the manufacturer's instructions. Z-stack confocal images were collected through the entire cells at 2µm intervals on an Eclipse Ti 2 inverted microscope (Nikon), equipped with a Nikon 60X/1,4_S oil immersion objective. Automatic counting of the PLA dots per image was performed using the 'Analyze particles' function in Image J software (5). All cells were analyzed within 5 images per treatment condition. The average cell number per image is: 17(SEM ± 1,28) for MDA-231 siCtrl and 14 (SEM ± 2,37) for MDA-231 siMemo1. Two-sided, unpaired t-test was used for statistical analysis. The technical negative controls are made by omitting Atox1 primary antibody and one of Memo1 and Atox1 secondary antibodies. Biological negative controls are made by detecting Atox1 and Beta Actin.

Wound healing assay. Cells were grown to confluency. 1×10^5 MDA-MB-231 cells were seeded in a tissue culture-treated 24-wells plate. Cells were left to attach to the plate bottom for 24 hours, at

which point adding siRNA for Memo1 silencing and siRNA for silencing control. Artificial wounds were created 24 h after silencing using a P-200 pipette tip to scratch on the cell monolayer. Refresh the medium for all wells. wound healing was monitored by bright field microscopy with a camera at 8, 12, and 24 hours. The area recovered by the migrating cells was calculated using ImageJ.

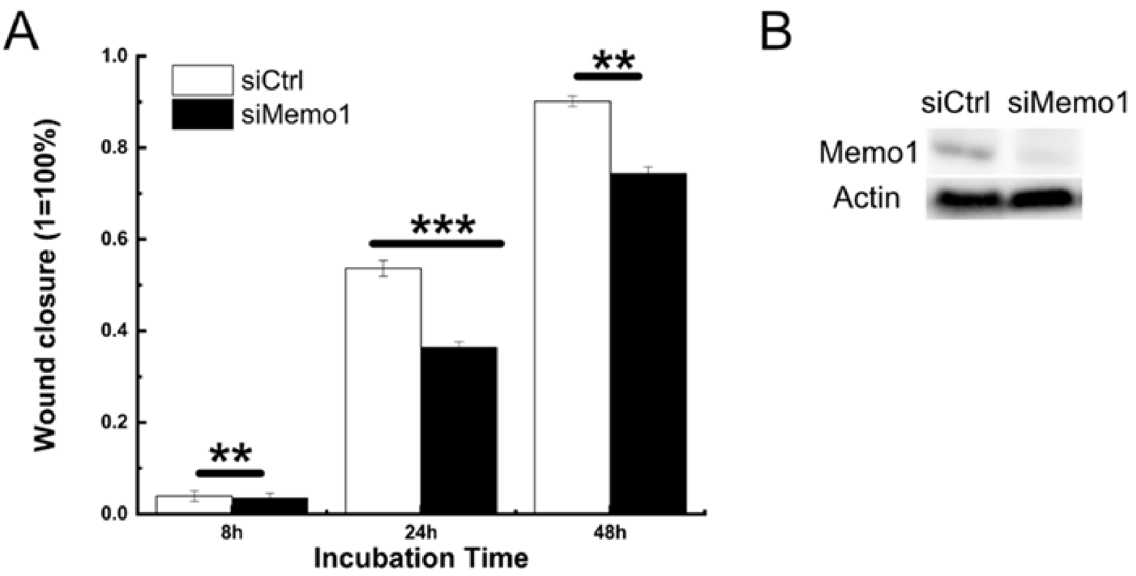

Fig. S1. (A) Memo1 downregulation reduces migration of MDA-MB-231 cells. Wound closure measured at 8 h and 24 h after wounding (error bar; standard error of the mean) ($n_{micrographs}$ = 10). ** 0.001 < p < 0.05; *** p < 0.001. (B) Western blot results of Memo1 knock down (siMemo1) and control cells (siCtrl) using Actin as control protein.

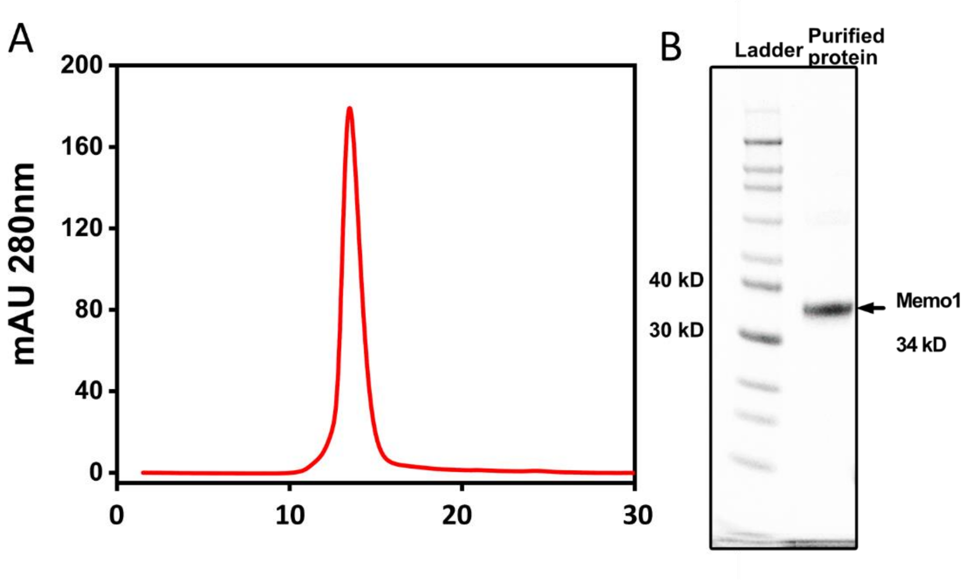

Fig. S2. (A) Elution profile of Memo1 after size exclusion chromatography through Superdex 75. (B) SDS PAGE and Coomassie staining of the purified protein.

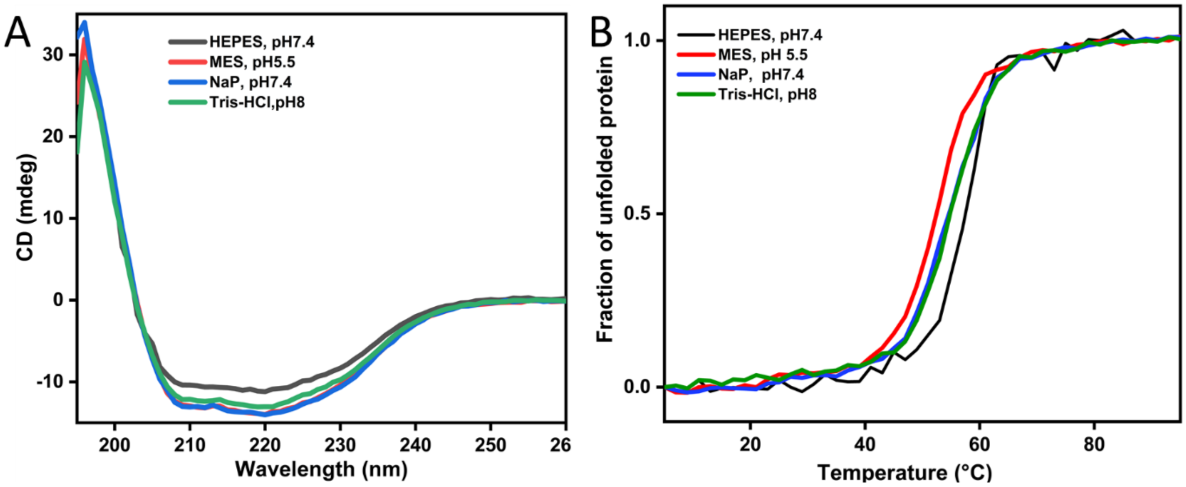

Fig. S3. Far UV CD spectra and thermal stability measurements of 5μM Memo1 in different buffers and pH. (A) Far UV (195-260 nm) spectra of Memo1 in different buffers and pH at 20°C. (B) Thermal unfolding profiles of 5μM Memo1 in different buffers and pH monitored at 222 nm.

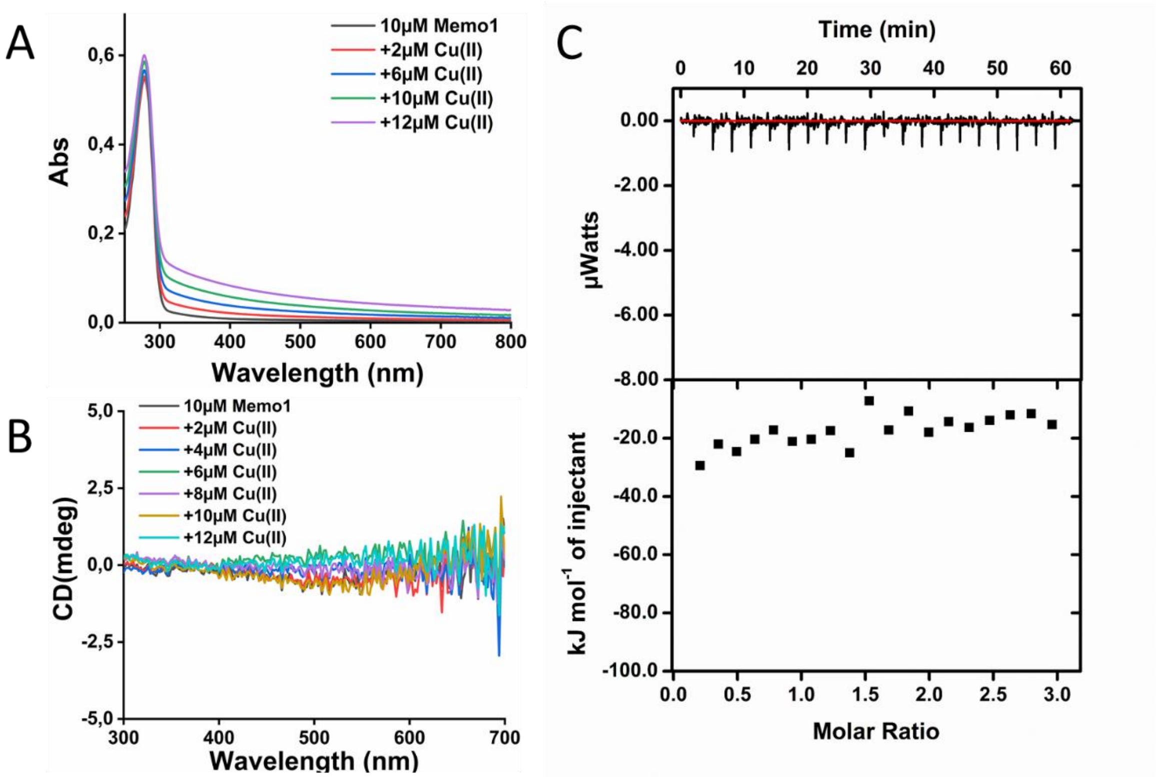

Fig. S4. No evidence for strong Cu(II) binding by Memo1. (A) The UV-visible spectra of Cu(II) titration to 10 µM Memo1. Note the appearance of scattering, indicative of protein aggregation with time and more Cu(II). (B) Visible CD spectra of Cu(II) titration to 10 µM Memo1. (C) ITC results for the Cu(II) titration to Memo1 (10 µM Memo1 in cell).

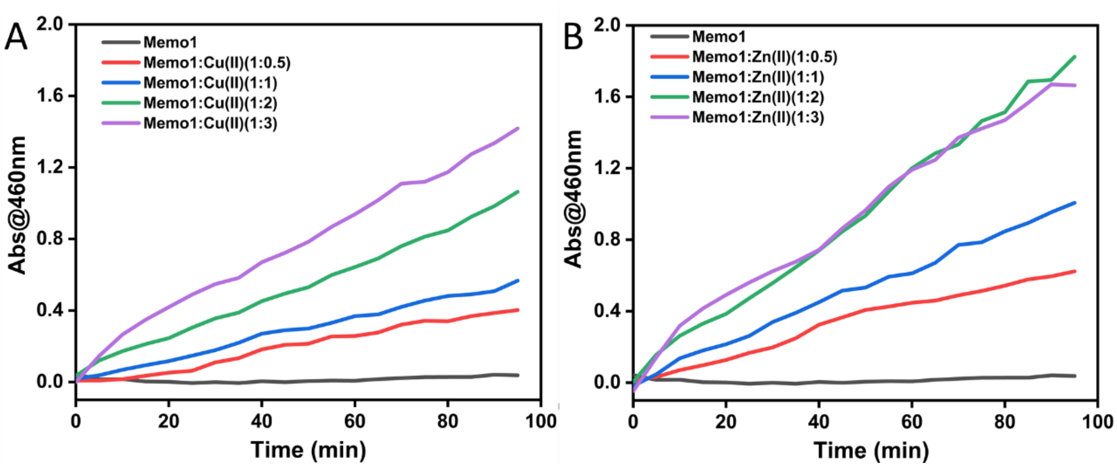

Fig. S5. Absorbance changes at 460 nm (reporting on scattering, i.e. protein aggregation) of 7 µM Memo1 as a function of time upon the additions of (A) Cu(II) and (B) Zn(II) concentrations from 3.5 to 21 µM.

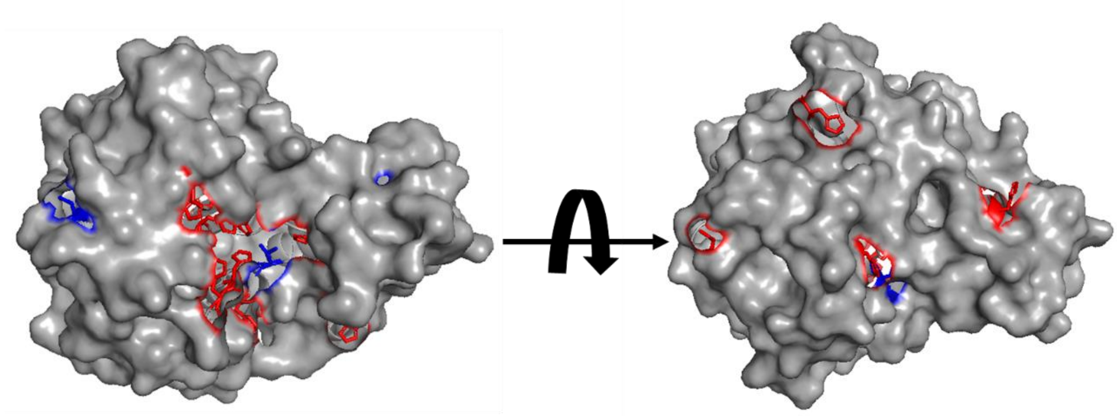

Fig. S6. Surface structure of Memo1 (PDB 3BCZ). His residues (H12, H36, H49, H61, H81, H82, H131, H147, H192, H215, H240, H247 and H297) are labeled as color red; Cys residues (C7, C55, C58, C88, C191, C244 and C279) are labeled as blue.

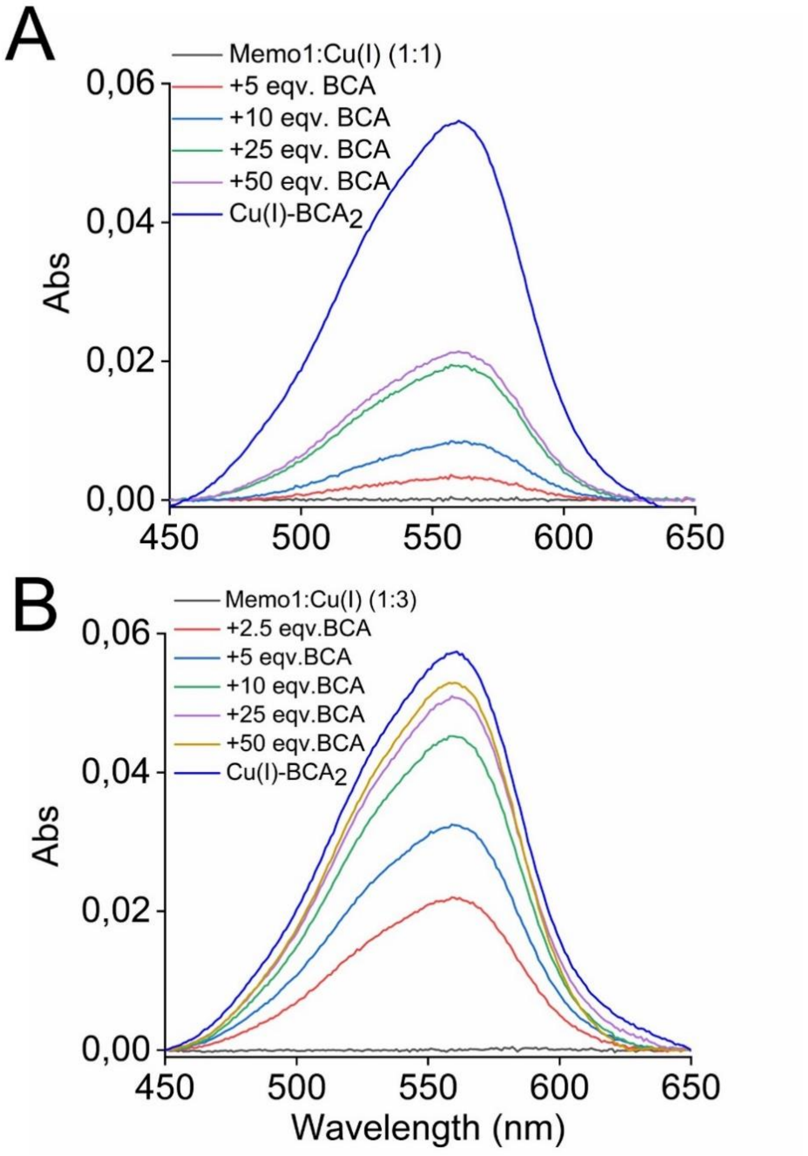

Fig. S7. Spectrophotometric analysis of Cu(I)-BCA_2 (10µM) and Memo1-Cu(I) (1:1 and 1:3 molar equivalents (eqv.) ratio) mixtures upon additions of BCA under anaerobic condition. (A) 50µM to 500µM BCA was added to 10µM Cu(I) pre-mixed with 10µM Memo1 (1:1). (B) 25µM to 500 µM BCA was added to 10µM Cu(I) pre-mixed with 3.3µM Memo1 (i.e., 3 to 1, Cu to Memo1 ratio). The experimental details are mentioned in Material and Methods.

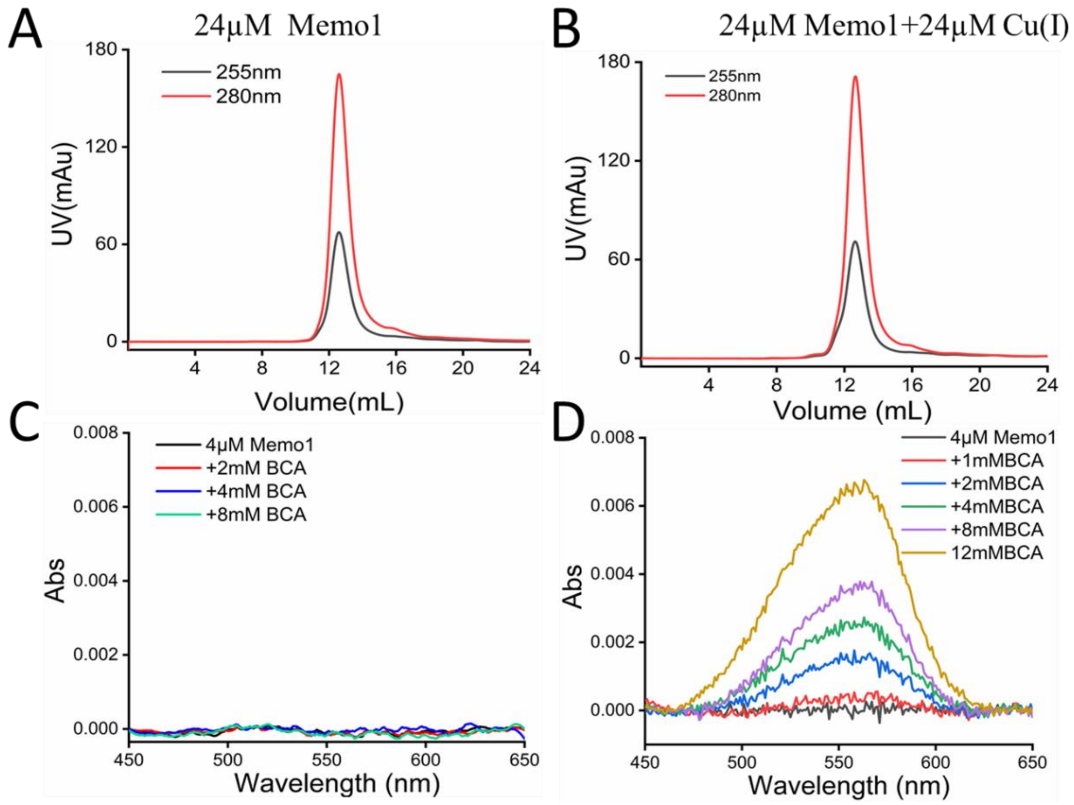

Fig. S8. Elution profiles of 24μM Memo1 without (A) and with 72μM DTT and 24μM Cu(II) (incubated at 4 ℃, 30 min) (B) from SEC showing absorbance at 255 nm (black) and 280 nm (red). The concentration of eluted Memo1 from (A) and (B) were 4μM. (C): Absorption spectra of BCA additions to 4μM eluted Memo1 from A. No Cu(I)-BCA_2 complex (absorption peak at 562nm) were detected. (D): Absorption spectra of BCA additions to 4μM eluted Memo1 from B. Absorption from Cu(I)-BCA_2 complexes was detected. Note that the titration could not be completed as additional BCA (above 12 mM; the 562 nm absorption value for 12 mM BCA corresponds to 1 μM Cu) precipitated the protein. Clearly the titration has not reached saturation and if more BCA could have been added, more Cu(I) would be detected (reaching 4 μM Cu). Nonetheless, this result still indicates that Memo1 can bind with Cu(I) since free Cu ions would have been removed from the protein during SEC. 1mM ascorbate acid were added to prevent Cu(I) oxidation prior BCA additions.

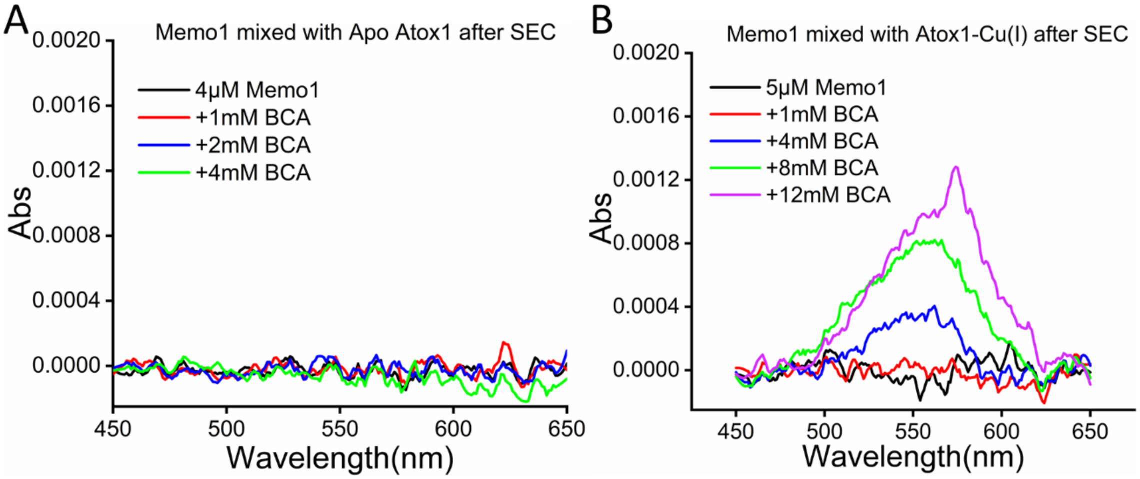

Fig. S9 Absorption spectra of BCA titration to eluted Memo1. **(A):** 24µM Memo1 were incubated with 50µM apo-Atox1 and 150µM DTT at 4 °C, 30mins, then the sample was loaded on SEC. The concentration of eluted Memo1 was 4µM. No Cu(I)-BCA_2 complex (absorption peak at 562nm) was detected upon addition of excess BCA. **(B):** 50µM Atox1 was pre-incubated with 150µM DTT and 50µM Cu, to form Cu(I)-loaded Atox1, at 4 °C for 30mins; then Cu-Atox1 was mixed with 24µM Memo1. After 30 min, the sample was loaded on SEC. The concentration of eluted Memo1 was 5µM. Upon titration with excess BCA, Cu(I)-BCA_2 complexes were detected, indicating that some Cu had moved from Atox1 to Memo1. The titration has not reached saturation, but additions of more BCA precipitated the protein. If more BCA could have been added to the Memo1 sample, more Cu(I) would likely be detected. The 562 nm absorption at 12 mM BCA corresponds to only 0.2 µM Cu, and we expect about 2.5 µM Cu to have transferred to Memo1 based on the amount of apo-Atox1 generated. It is possible that Memo1 loses some Cu(I) during the column separation which is not at anaerobic conditions. ICP-MS analysis of eluted Memo1 from **B** also detected sub-micromolar levels of Cu ions (low but significantly higher than negative controls). To note, ICP-MS of pure Memo1-Cu(I) samples also includes some metal ion losses (**Table S1**).

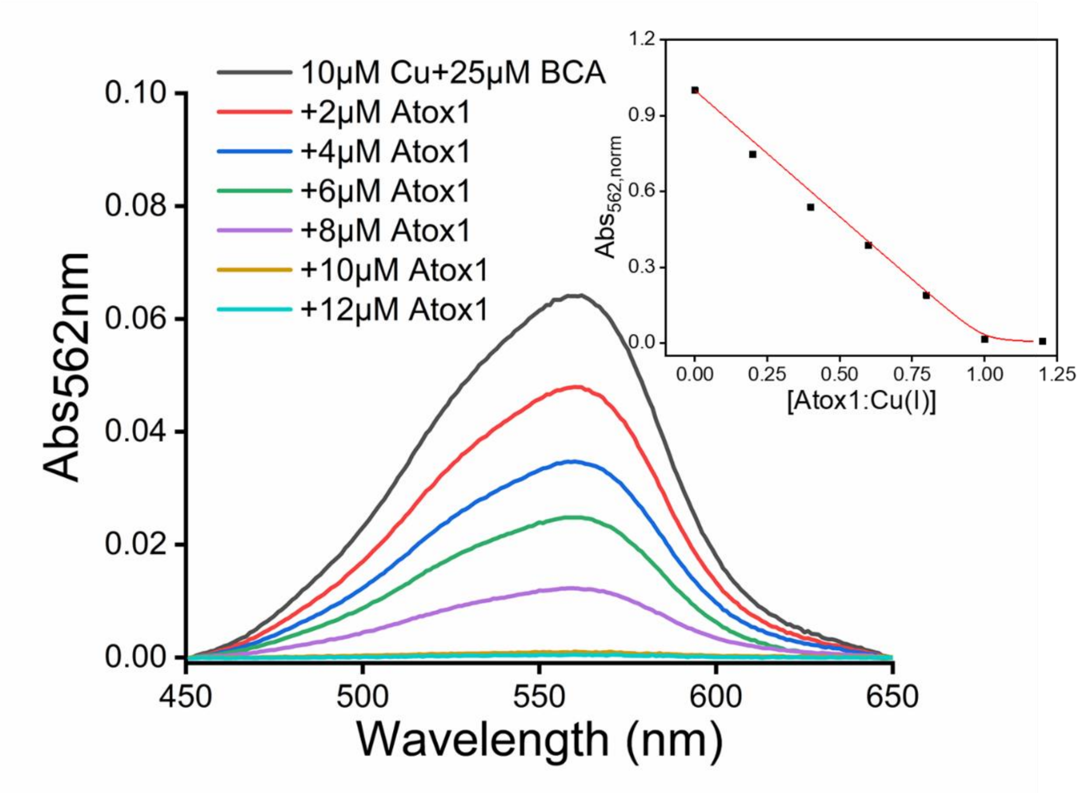

Fig. S10. Cu(I)-BCA_2 competition assay with Atox1 at strict anaerobic conditions. Absorption spectra of 2 to 12µM Atox1 (in 25mM HEPES buffer pH 7.4 with 200mM NaCl) titrated to 10µM Cu(I)-BCA_2 complex. Insert shows best fit to Eq.2 of absorbance changes at 562nm. A K_D for Cu(I) to Atox1 is 6.3×10^{-17} M which is similar to values found in the literature (2).

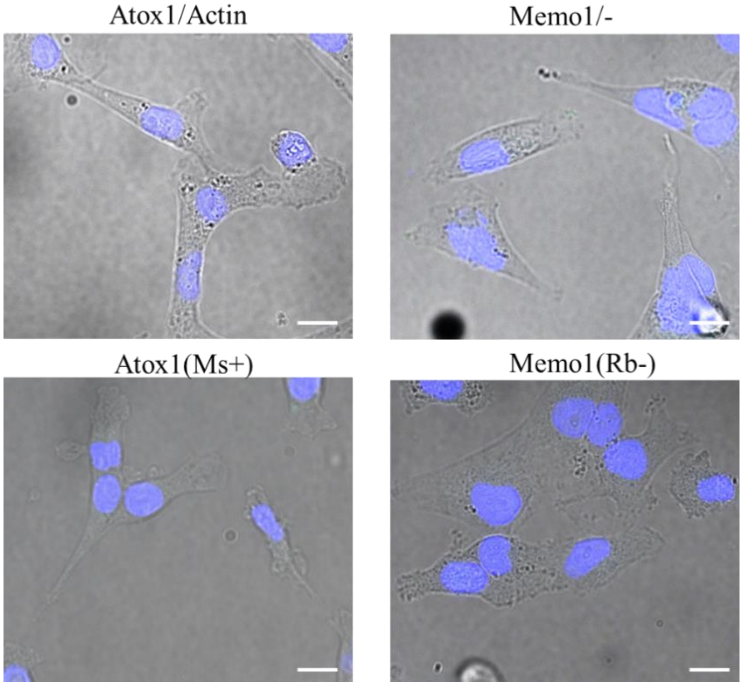

Fig. S11 Proximity ligation assays (PLA) technical (omit Atox1primary antibody or omit one of the secondary antibodies) and biological (Atox1-Beta Actin) negative controls. Representative maximum projection confocal micrographs (blue=DAPI, indicating nuclei; green=PLA dots) merged with phase contrast images. Scale bars indicate 10 μm.

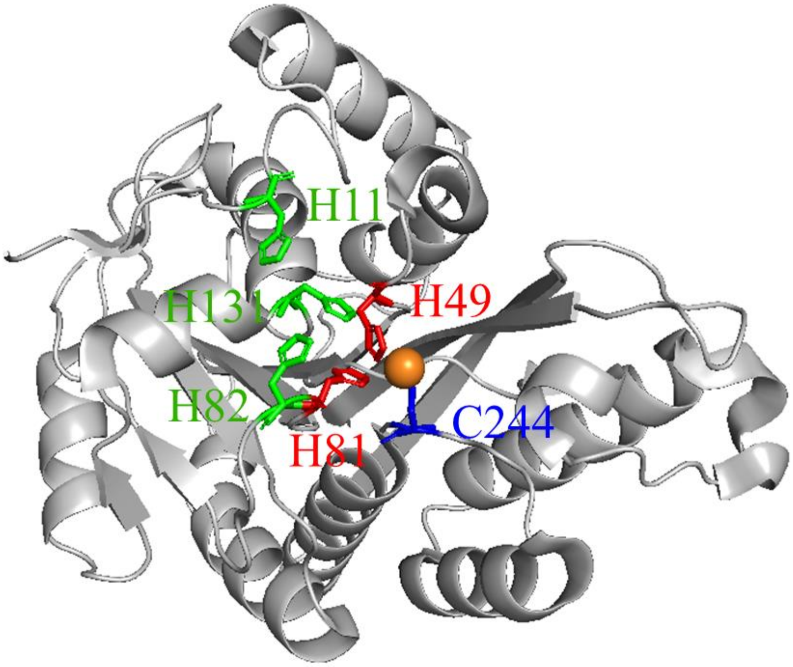

Fig. S12 Ribbon structure of Memo1 (pdb 7L5C), showing the proposed Cu(I) (brown sphere) binding site, with side chains of His49, His81 and Cys244 in red and blue stick representation (6). Upon inspection, we identified another putative Cu(I) binding site involving side chains of His12, His82 and His131 in green stick representation (these residues coordinate a water molecules in the crystal structure that was collected at pH 5).

Table S1. ICP-MS data showing Cu associated with Memo1 protein that are removed from the coordination of BCA ligand.

Samples	[Cu](µM)	[Cu] error (µM)
9.5µM MEMO1 (10µM Cu(I)-BCA_2 + 10µM Memo1 (1:1))	4.9	0.040
9.5µM MEMO1 (5µM Cu(I)-BCA_2 + 10µM Memo1 (1:0.5) mixed followed by dialysis)	2.7	0.0025
25mM HEPES buffer pH 7.4 with 200mM NaCl + 3µM Cu(II)	2.9	0.020
25mM HEPES buffer pH 7.4 with 200mM NaCl	0.041	0.0017

SI References

1. M. E. Palm-Espling, C. D. Andersson, E. Bjorn, A. Linusson, P. Wittung-Stafshede, Determinants for simultaneous binding of copper and platinum to human chaperone Atox1: hitchhiking not hijacking. *PLoS One* **8**, e70473 (2013).
2. Z. Xiao *et al.*, Unification of the copper (I) binding affinities of the metallo-chaperones Atx1, Atox1, and related proteins: detection probes and affinity standards. *Journal of Biological Chemistry* **286**, 11047-11055 (2011).
3. D. Kahra, M. Kovermann, P. Wittung-Stafshede, The C-Terminus of Human Copper Importer Ctr1 Acts as a Binding Site and Transfers Copper to Atox1. *Biophys J* **110**, 95-102 (2016).
4. S. Blockhuys, X. Zhang, P. Wittung-Stafshede, Single-cell tracking demonstrates copper chaperone Atox1 to be required for breast cancer cell migration. *Proc Natl Acad Sci U S A* **117**, 2014-2019 (2020).
5. J. Schindelin *et al.*, Fiji: an open-source platform for biological-image analysis. *Nature methods* **9**, 676-682 (2012).
6. N. a. U. Dolgova, Eva-Maria E. and Boniecki, Michal T. and Vizeacoumar, Frederick S. and Ralle, Martina and Tonelli, Marco and Abbas, Syed A. and Patry, Jaala and Elhasasna, Hussain and Freywald, Andrew and Vizeacoumar, Franco J. and Dmitriev, Oleg Y. (2022) MEMO1 Is a Metal Containing Regulator of Iron Homeostasis in the Cell. http://dx.doi.org/10.2139/ssrn.4084075.
